# Sphingosine Kinase 1 Deficiency Exacerbates LPS-Induced Neuroinflammation

**DOI:** 10.1371/journal.pone.0036475

**Published:** 2012-05-17

**Authors:** Natalia M. Grin’kina, Eddy E. Karnabi, Dushyant Damania, Sunil Wadgaonkar, Ilham A. Muslimov, Raj Wadgaonkar

**Affiliations:** 1 SUNY Downstate Medical Center, Brooklyn, New York, United States of America; 2 Department of Research and Development VA Medical Center, Brooklyn, New York, United States of America; University of North Dakota, United States of America

## Abstract

The pathogenesis of inflammation in the central nervous system (CNS), which contributes to numerous neurodegenerative diseases and results in encephalopathy and neuroinflammation, is poorly understood. Sphingolipid metabolism plays a crucial role in maintaining cellular processes in the CNS, and thus mediates the various pathological consequences of inflammation. For a better understanding of the role of sphingosine kinase activation during neuroinflammation, we developed a bacterial lipopolysaccharide (LPS)-induced brain injury model. The onset of the inflammatory response was observed beginning 4 hours after intracerebral injection of LPS into the lateral ventricles of the brain. A comparison of established neuroinflammatory parameters such as white matter rarefactions, development of cytotoxic edema, astrogliosis, loss of oligodendrocytes, and major cytokines levels in wild type and knockout mice suggested that the neuroinflammatory response in SphK1−/− mice was significantly upregulated. At 6 hours after intracerebroventricular injection of LPS in SphK1−/− mice, the immunoreactivity of the microglia markers and astrocyte marker glial fibrillary acidic protein (GFAP) were significantly increased, while the oligodendrocyte marker O4 was decreased compared to WT mice. Furthermore, western blotting data showed increased levels of GFAP. These results suggest that SphK1 activation is involved in the regulation of LPS induced brain injury.

**Research Highlights:**

• Lipopolysaccharide (LPS) intracerebral injection induces severe neuroinflammation. • Sphingosine kinase 1 deletion worsens the effect of the LPS. • Overexpression of SphK1 might be a potential new treatment approach to neuroinflammation.

## Introduction

The inflammatory response starts with recognition of viral or bacterial products and typically plays a defensive role [1], [2], but when these mechanisms are overactive the evoked cascade can be fatal [3]. In the central nervous system, inflammation with a bacterial etiology is thought to contribute to numerous neurological disorders, including Alzheimer disease [4], [5], Parkinson disease [6], periventricular leukomalacia [7], multiple sclerosis [8], [9], head trauma, spinal cord injury [5] and sepsis [10]. During neuroinflammation as a first line of defense, the innate immune system recognizes a limited number of invading pathogens. Among them is lipopolysaccharide (LPS), an endotoxin which forms most of the outer membrane of gram-negative bacteria [11]. It has been shown that LPS binding to toll-like receptors (TLR’s) and clusters of differentiation (CD11 and CD14) [2] activates a variety of immune cells such as monocytes, macrophages, leukocytes and neutrophils. In the mammalian immune system, lipopolysaccharide is recognized by toll-like receptor-4 (TLR4) [12]. Intracerebral injection of LPS in TLR4−/− mice did not result in neutrophil activation [13], and mice lacking this receptor were hyporesponsive to LPS in general [14].

Sphingosine-1-phosphate (S1P) is a bioactive sphingolipid shown to be a key player in cellular lipid metabolism; it also serves as an important signaling molecule [15], [16]. By binding to its receptors, S1P regulates several physiological functions, including angiogenesis, vascular permeability, lymphocyte recirculation, cell differentiation, proliferation, cytokine/chemokine generation and apoptosis [17], [18], [19], [20]. S1P is generated by the phosphorylation of sphingosine by sphingosine kinase [17]. There are two types of sphingosine kinases, type 1 and type 2 (SphK1 and SphK2) [20]. The catalytic domain structure is similar in both kinases, however SphK2 has additional domains including a nuclear localization signal (NLS) domain which is present in the N-terminus of SphK2, as well as a pro-apoptotic BH3 domain and lipid binding domain [21], [22].

In a previous study we demonstrated that in the lung vasculature, the activation of SphK1 and subsequent S1P generation plays a barrier-protective role by controlling inflammatory responses [23]. We reported a differential role of the SphK1 and SphK2 in lipid raft signaling and lung injury. In an LPS induced lung injury model, SphK1−/− mice were much more susceptible to injury compared to their WT counterparts, as quantified by multiple parameters including cytokine induction. Of particular interest, overexpression of WT SphK1 delivered by adenoviral vector to the lungs protected SphK1−/− mice from lung injury, and attenuated the severity of the response to LPS. However, adenoviral overexpression of a SphK kinase-dead mutant, which can also block SphK2 activity in SphK1−/− mice, further exacerbated the response to LPS as well as the extent of the lung injury. This suggested that in vascular injury, activation of SphK1 plays a protective role. In SphK1−/− mice, we have further shown that LPS induced lung injury was significantly reduced by S1P injection. Earlier studies by Garcia [24], Peng [25] and McVerry [26], [27] have shown that S1P is barrier protective, and in different models S1P treatment increased protection against lung injury.

Recently, the role of both SphKs in immunity and inflammation has been the main focus of multiple studies [28], [29], [30], [31]. However, these studies are conflicting, suggesting a broader role for sphingosine kinases in vascular injury. Puneet‘s study published that inhibition of Sphk1 led to decreased phagocyte production of endotoxin-induced proinflammatory cytokines [32]. Other studies showed that inhibition of SphK1 by its inhibitor and/or siRNA decreased expression of proinflammatory cytokines, such as TNFα, IL-1β and iNOS, when activated with LPS, microglia cells released these chemokines [33].

In our study that involved genetically altered SphK1−/− animals, we hypothesize that in the sphingolipid rich environment of the brain; deletion of SphK1 will have a deleterious effect on the development of inflammation. To test our hypothesis, brain injury was induced by direct injection of LPS (1 mg/kg) into the lateral ventricles. In this study, we demonstrate that SphK1−/− mice are more vulnerable to the inflammation and injury induced by LPS. These findings suggest that SphK1 plays a crucial role in protection from the development of neuroinflammation induced by intracerebral LPS injection.

## Materials and Methods

### Animal Protocol

All animal protocols were approved by the Institutional Animal Care and Use Committees of the Veterans Affairs (VA) Medical Center and State University of New York (SUNY) Downstate Medical Center (Protocol **ID** # 01165; **Prom#:** 0002).

### Genotyping of SphK1 Mutant and Wild Type Mice

Genotyping for the SphK1 alleles was determined by PCR analysis of genomic mouse DNA isolated from tail biopsies. Total DNA was extracted by conventional methods and was used as the template (40 cycles of 94°C for 1 min, 60°C for 1 min, and 72°C for 1 min with the extension at 72°C for 7 min) with neomycin cassette forward SphK1 primer (5_TCGTGCTTTACGGTATCGCCGCTCCCGATT_3), reverse SphK1 primer (5_AGAAGGCACTGGCTCCAGAGGAACAAG_3), forward wild type primer (5_TGTGGTGGTGTTGTGTTTTGTTTGTAGT_3) and reverse wild type primer (5_AGCATAGTGGTTCACAGAAGCTGCCA_3). The expected product sizes for the wild-type and targeted SphK1 alleles were 500 bp and 340 bp, respectively.

### Animal Protocol Methods

SphK1−/− mice and their counterpart C57BL/6 WT controls were selected for procedures. 6–12 month old (n = 96) of both sexes were used for the intracerebroventricular injection. All mice were divided into 4 groups (24 animals per group): wild type saline injected, wild type LPS injected, SphK1−/− saline injected, and SphK1−/− LPS injected. LPS from E.coli serotype 0127:B8 (Sigma-Aldrich) was used to induce brain injury, and sterile saline was used as a control. Mice were anesthetized with a mixture of ketamine (100 mg/kg) and xylazine (10 mg/kg) diluted in sterile saline and were placed in a stereotaxic apparatus. Intracerebroventricular injection was performed as previously described [34]. Briefly, a skull incision was made, exposing the bregma. At a location of AP – (−1.0 mm), ML – (1.0 mm), DV – (2 mm) to the bregma, LPS was administered at a dose of 1 mg/kg body weight. Control mice were injected in the same location with sterile saline. Injected volumes were 2.5 µl each. The injection was completed within 5 minutes, at speed 0.2 µl/min; the glass pipette was kept in the position for an additional 2 minutes following injection, and then slowly extracted. The incision was sutured and mice were kept at 37°C until recovery. All animals survived the intracerebroventricular injection. Six hours after the injection, mice were sacrificed either by cervical dislocation and decapitation for brain tissue preparation for Western Blot, or by transcardiac perfusion with 0.1 M PBS followed by freshly prepared 4% paraformaldehyde, 10% sucrose and further incubation of extracted brains in increasing gradient sucrose solution. 8 micron thick frozen coronal cryosections of the brain were prepared for immunohistochemical and histological analysis.

### Immunohistochemistry and Histological Examination

For immunohistochemistry, sections were prepared as previously described [7]. Hematoxylin and Eosin (H&E) staining and immunohistochemistry (IHC) were performed in consecutive frozen sections (8 microns). H&E sections were examined using an upright epifluorescent microscope for any alterations in morphology. The area of the brain at the level of bregma: 0.74–1 mm and interaural: 4.39–5 mm was analyzed. Sizes of both lateral ventricles were measured as areas of a triangle (A = ½ of the base x height) (Fig. S1). Total area of the brain section was taken as a 100 percent and percent taken by lateral ventricles was calculated.

White matter rarefactions were blindly counted on non-adjacent sections that had intact cytoarchitechture. Analysis was performed on the ipsilateral side of the injection. For immunohistochemistry, sections were incubated in a fixative solution of 4% paraformaldehyde overnight at 4°C, followed by incubation in 0.3 M glycine in PBS for 10 minutes to remove autofluorescence. Sections were then blocked in a buffer containing 10% normal goat serum, 0.5% BSA and 0.5% Triton X-100 in PBS for 1 hour at room temperature. Rinsed sections were incubated overnight at 4°C with primary antibody diluted at different ratios in 1% normal serum, 0.5% BSA and 0.5% Triton X-100 in PBS at 4°C. In order to detect reactive astrocytes, GFAP antibody (1∶300) (Cell Signaling Technology, Inc., Danvers, MA) was used; to visualize oligodendrocytes O4 antibody (1∶200) (Sigma Aldrich, St.Louis, MO) was applied; to determine reactive microglia Ferritin Light Chain antibody (1∶50) (SantaCruz, Santa Cruz, CA) and/or CD68 antibody (1∶100) (SantaCruz, Santa Cruz, CA) were used. After washing, sections were incubated with FITC and/or TRITC conjugated secondary antibodies (1∶1000) (Sigma Aldrich, St.Louis, MO) in the dark for 2 hours at room temperature. To make sure that gain or loss of staining was not due to higher/lower number of cells, DAPI (Sigma Aldrich, St.Louis, MO) and/or propidium iodide stain was applied in order to visualize nuclei of all cells in the studied area of the mouse brain. Sections were then washed, dried, mounted, and studied using an upright compound epifluorescent microscope. Threshold images of series’ of consecutive sections were analyzed using NIH published software ImageJ. Areas for protein expression quantifications were chosen stereotactically and based on the *in situ* hybridization database of Allen institute of brain science (http://www.brain-map.org/). Expression of astrocytes’ glial fibrillary acidic protein (GFAP), microglial CD68 and ferritin light chain protein was quantified on the ipsilateral side of the injection at the septal complex area. Oligodendrocytes progenitor cells O4 protein expression was quantified at the area of lateral piriform cortex on the ipsilateral side of the injection.

### Proteins Extraction and Western Blot Technique

Membrane proteins were prepared using the following protocol [35]. Brains were harvested and dissected. The tissue was weighed and homogenized in RIPA buffer (50 mM Tris HCl pH = 7.4; 150 mM NaCl; 1 mM PMSF; 0.1%SDS; 1% Triton X-100; 1% Sodium Deoxycholate) complete with protease inhibitor cocktail (Sigma Aldrich, St Louis, MO). Homogenization was performed in short 10 second bursts followed by 5 minute intervals on ice. Cytoplasmic and nuclear fractions were separated by centrifugation, followed by determination of protein concentration in triplicate by a Bio-Rad DC protein assay kit (BioRad, Hercules, CA). Equal concentrations (30 µg) of proteins were loaded in each lane of a standard 4–12% SDS polyacrylamide gel. After electrophoresis, proteins were transferred to a PVDF membrane (GE Healthcare Life Science, Piscataway, NJ), then blocked in 0.1 M PBS containing 0.1% Tween (PBS-T) and 5% non-fat milk for 1 hour. The membranes were then incubated overnight in a primary antibody specific for the protein of interest at 4°C. Antibodies were diluted at 1∶1000–1∶10000 ratio in PBS-T solution containing 0.5% milk. After washing, the immunoblots were incubated with the appropriate horseradish peroxidase-labeled secondary antibodies (1∶2000) in PBS-T for 2 hours at room temperature. Monoclonal GFAP antibody was purchased from Cell Signaling Technology, Inc., Danvers, MA. The final step was detection by ECL according to manufacturer’s instructions (Amersham Biosciences, Pittsburgh, PA). Integrated densities of the bands were quantified using Image J software and calculated as a ratio of absolute intensity to the intensity of the background.

### Statistical Analysis

Data analysis was performed using unpaired two-way ANOVA or non-parametric Wilcoxon test when appropriate; Bonferroni’s multiple comparison of the groups was used to test the effects of the treatment and/or of the gene. Data is presented as means ±SEM. A value of p≤0.05 is considered significant.

## Results

### SphK1−/− Mice Exhibit Severe Edema and Bigger White Matter Loss as a Result of Intracerebral LPS injection, Compared to Wild Type

**Figure 1:**
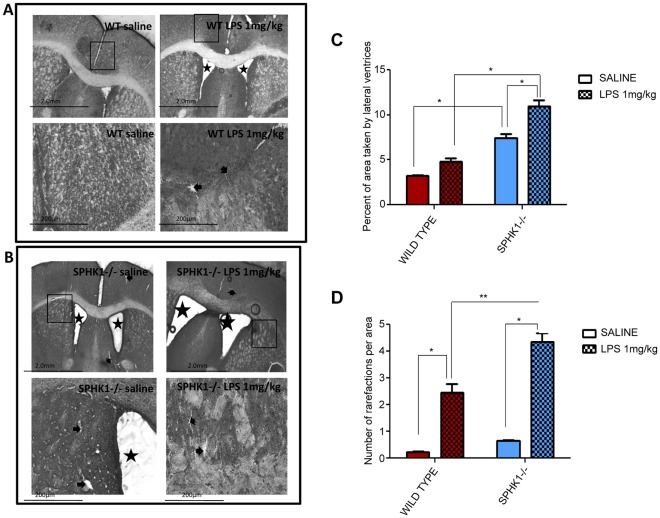
Hematoxylin and eosin staining of the frozen sections of the SphK1−/− type mice. (**A**) Wild type mice underwent the intracerebral injection of either saline or LPS 1 mg/kg for 6 hours. Frozen sections of the brains were prepared and stained with hematoxylin and eosin. LPS injected mice exhibit enlarged ventricles (stars) and white matter rarefactions (arrows), two of the neuroinflammation marker. Magnification of 4×, 10× and 20× are shown. (**B**) Hematoxylin and eosin staining of the frozen sections of the SphK1−/− type mice. Enlarged ventricles (stars) and white matter rarefactions (arrows) were observed in control SphK1−/− animals, these neuroinflammatory parameters were increased after LPS injection. Magnification of 4×, 10× and 20× are shown. (**C**) Two-way ANOVA statistical analysis of the increase of the sizes of the lateral ventricles. Injection of LPS 1 mg/kg into lateral ventricle of the brain significantly (p<0.0001) affected development of the edema in SphK1−/− group. When Bonferroni’s multiple comparison was performed, statistical significance was reached between similarly treated, but genetically different groups, such as wild type LPS 1 mg/kg vs. SphK1−/− LPS 1 mg/kg (*p<0.0001); wild type saline vs. SphK1−/− saline (*p<0.0001). (**D**) Statistical analysis of the loss of the white matter in the brains of the animals intracerebroventicularly injected with saline or LPS 1 mg/kg. Statistical significance was reached after the injection of LPS 1 mg/kg in wild type and SphK1−/− mice (*p<0.0001). SphK1 deletion had a significant effect (**p<0.0001) in the development of leukoaraiosis.

Direct lipopolysaccharide intracerebroventricular injection leads to much worse neuroinflammatory reactions in the brain, although it does relax the blood brain barrier function through loosening of tight junctions when injected intraperitoneally [36], [37]. During neuroinflammation, changes in vascular permeability results in the development of brain edema. Hematoxylin and eosin (H&E) stained coronal brain sections were carefully studied under light microscope for any pathological changes. The difference in the responses to treatment between wild type and SphK1−/− mice was prominent. In the central nervous system, the above mentioned neuroinflammatory processes lead to the enlargement of the lateral ventricles of the brain (Fig. 1A and 1B). The percent of the area of the lateral ventricles per brain section was measured and statistically analyzed (Fig. 1C). SphK1−/− LPS (1 mg/kg) injected mice had the largest ventricles among the four groups of experimental animals, with 10.98±0.6177 (11%) in this group significantly (p<0.0001) higher than in SphK1−/− saline injected 7.44±0.4234 (7.2%) and 2 folds higher than in WT LPS (1 mg/kg) injected mice 4.8±0.4057 (Fig. 1C). To our interest, SphK1−/− saline injected animals expressed some enlargement of the ventricles too (7.44±0.4234), which was significant compared to wild type saline injected group (3.219±0.12) (*p<0.0001). These findings support our hypothesis that SphK1−/− animals are more susceptible to the injury and that deletion of it affects the baseline response to LPS. Leukoaraiosis is a loss of white matter that is usually associated with vascular risk factors such as hypertension, or in the context of cognitive impairment [38]. On the other hand, there are several reports where leukoaraiosis was found to be in a close relationship with the inflammatory markers [39] and loss of oligodendrocytes, followed by demyelination [40]. We calculated the percent of observed white matter rarefactions per taken cortex area. The process of leukoaraiosis was doubled in SphK1−/− LPS treated animals (4.4±0.33) compared to wild type LPS treated group (2.4±0.33), **p<0.0001 (Fig. 1D). The fold increase between saline and LPS (1 mg/kg) injected animals in the wild type group (10 folds) was almost 2 times bigger than that in knockout group (6.8 folds) (Fig. 1D). This effect is possibly due to the presence of the considerable amount of rarefactions observed in SphK1−/− control mice (0.6±0.04) which is significantly higher than that in wild type saline treated animals (0.229±0.03311). Nevertheless, the total number of rarefactions in wild type LPS treated animals was (2.4±0.33), which we believe is partially due to the presence of sphingosine kinase 1 and its pro-survival effects.

Observed rapid increases in the sizes of lateral ventricles and the basal differences between wild type saline injected and SphK1−/− saline injected animals led to the analysis of these parameters in naïve animals. Statistically significant differences were found between the sizes of the lateral ventricles of wild type and SphK1−/− (*p = 0.0008) animals (Fig. S2). Brain matter of SphK1−/− animals appeared to be more distorted (Fig. S2), pointing to the role of SphK1 not only in the exacerbation of the neuroinflammatory processes, but in neurogenesis as well. These observation of ventricular enlargement and white matter rarefactions are consistent with the observations of Tauseef et al., who demonstrated an enhanced pulmonary edema in SphK1−/− animals in response to LPS [41]. The most interesting finding of our experiment was the presence of enlarged lateral ventricles in mutant mice who received saline injection as a control; leading us to consider the possibility of SphK1 as being an important molecule in the mechanism of neurogenesis, but this observation needs to be explored further.

### SphK1−/− Animals have More Reactive Microglia after Intracerebral Injection of LPS

**Figure 2:**
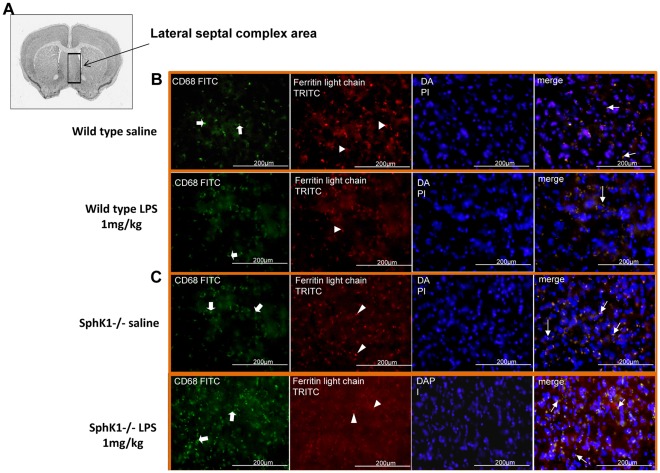
Immunohistochemistry of brain sections after intracerebroventricular LPS injection. (**A**). Lateral septal complex area of the brain was chosen for the analysis based on *in situ* hybridization database. Interaural: 4.39 mm: bregma: 0.74 mm. (**B**). Immunohistochemistry of the frozen sections of the brains of wild type mice that received intracerebroventricular injection of saline or LPS 1 mg/kg. Reactive microglia was visualized by double staining with anti-CD68 (FITC – thick arrows) and anti-ferritin light chain (TRITC – arrow heads) antibody. DAPI stain was used to detect nuclei of all cells. Magnifications of 40× are shown. (**C**). Anti-CD68 (FITC), anti-ferritin light chain (TRITC) positive staining and DAPI staining of the frozen brain sections of SphK1−/− experimental animals. Magnifications of 40× are shown.

**Figure 3:**
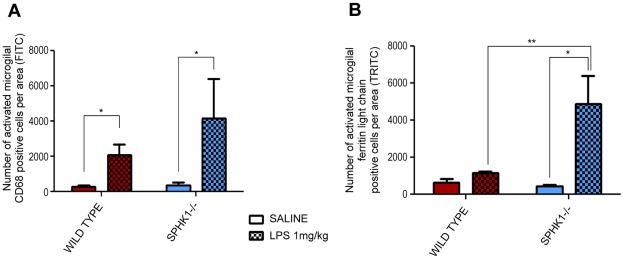
Immunohistochemistry using anti- CD 68 and ferritin light chain antibodies. (**A**) Two-way ANOVA analysis revealed significant difference (*p = 0.0451) in anti-CD68 (FITC) staining after LPS 1 mg/kg intracerebroventricular injection in wild type and SphK1−/− mice. (**B**) Bonferroni’s multiple comparison of the anti-ferritin light chain staining (TRITC) determined significance between wild type LPS 1 mg/kg vs. SphK1−/− LPS 1 mg/kg (**p = 0.0181) and in mutant group after LPS injury (*p = 0.0123).

Since microglia are considered to be the macrophages of the brain, in the next experiment we studied reactive microglia that are highly expressed during neuroinflammation. Resident microglia of the brain become reactive following various forms of insult, including chemical trauma and viral and/or bacterial infection. We used two types of the specific antibodies to detect reactive microglia: anti-ferritin light chain [42], [43] and anti-CD68, which are clusters of differentiation that are heavily expressed on the surface of macrophages and microglial cells across the brain. The interaction of the antibody and proteins was visualized using secondary antibodies conjugated to TRITC and FITC, respectively (Fig. 2B and Fig. 2C). The lateral septal complex brain area was chosen for reactive microglia immunohistochemical study (Fig. 2A). When CD68-positive cells were quantified and the data analyzed, significant increases were only found in wild type and SphK1−/− animals treated with LPS (1 mg/kg) (*p = 0.0451) (Fig. 3A). Statistical analysis of the images of the immunohistochemically stained sections with anti-ferritin light chain antibody showed that treatment with LPS (1 mg/kg) only had a significant effect (*p = 0.0123) in the SphK1−/− group (Fig. 3B). In addition, statistical interaction was found (p = 0.0357) between groups. In proof of our hypothesis that deletion of SphK1 has a devastating effect in the development of the inflammatory processes in the brain, Bonferroni’s multiple comparison of the groups displayed a very significant effect between wild type LPS 1 mg/kg and SPHK1−/− LPS 1 mg/kg group (**p = 0.0181), where the difference was more than 5 folds in the numbers of ferritin-light chain positive microglia (Fig. 3B).

### Mild Astrogliosis in Wild Type mice as Compared to SphK1−/− Mice

**Figure 4:**
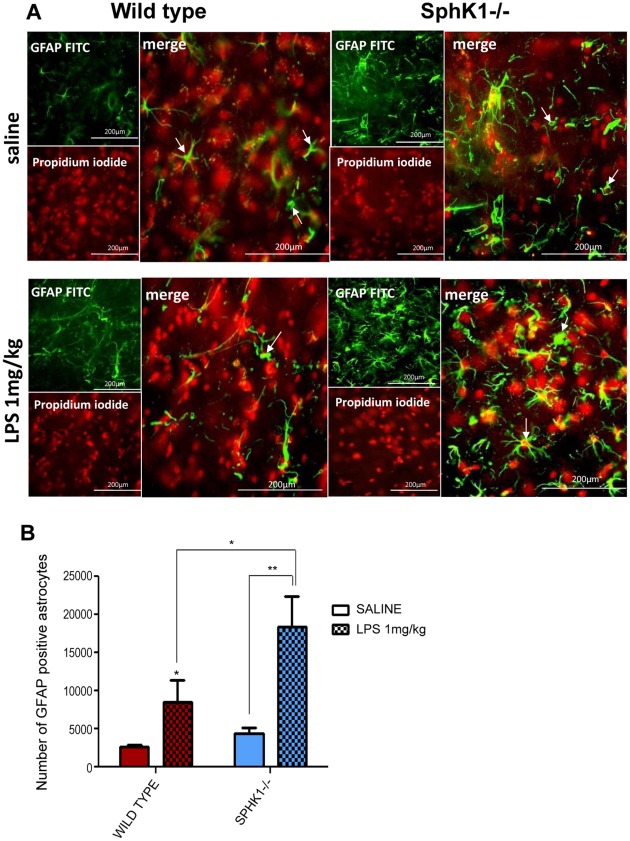
(A) Immunohistochemistry of the frozen sections of the brains. GFAP specific antibody were used as a marker of reactive astrocytes (arrows), propidium iodide was used as a marker of all cells in the studied lateral septal complex area of the animals’ brain. SphK1−/− mice after LPS induction expressed the most of reactive astrocytes. Magnifications of 40× are shown. (**B**) Reactive astrocytes were quantified and statistically analyzed using two-way ANOVA. Significant results were reached in SphK1−/− groups after treatment with LPS 1 mg/kg (*p = 0.0150). SphK1−/− LPS 1 mg/kg injected animals expressed the highest number of the reactive astrocytes.

Astrogliosis is another marker of neuroinflammation [44]. Therefore, our next step was to determine the extent of gliosis in wild type and SphK1−/− mutant mice. Astrogliosis is an appearance of gemistocytes, which are reactive astrocytes with enlarged cytoplasm and proliferating processes [45]. Immunohistochemical analysis using the astrocyte specific antibody GFAP was performed. Wild type and SphK1−/− mice reacted vigorously to the intracerebral injection of LPS (1 mg/kg) by expressing more reactive astrocytes, as compared to saline treated controls (Fig. 4B). Stained slides were inspected under epifluorescent microscope, with close attention paid to the lateral septal complex area, shown in Figure 2A. The quantitative analysis of a series of thresholded 40× magnified images was performed. Wild type mice exhibited an increase in reactive astrocytes from 2603±284.4 in controls to 8521±2918 in LPS treated animals (*p<0.05) (Fig. 4B). In SphK1−/− LPS injected mice, reactive astrocytes were calculated at 18370±3972 per taken area, which was significantly higher (**p<0.0150) than in saline injected group (4412±722) and any wild type group.

**Figure 5:**
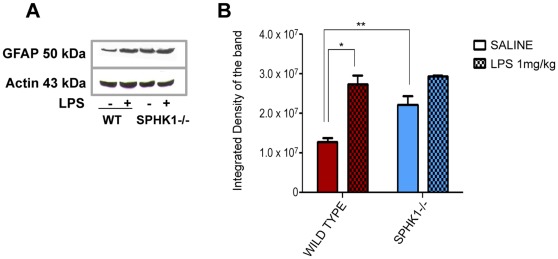
(A) Western blot analysis of the GFAP protein expressed in the total brain extract. (**B**) Statistical analysis of the integrated densities of the bands of the western blot. Significance was reached in wild type group after LPS induction (*p<0.0001) and in saline injected group between wild type and SphK1−/− animals (**p<0.001).

When Western blotting was performed using the GFAP antibody (Fig. 5A) a significant difference was observed in wild type group between saline and LPS (1 mg/kg) injected animals (*p<0.0001). SphK1−/− saline injected mice exhibited very strong expression of GFAP, which was significantly different from wild type saline animals (**p<0.001), but no significance was found in SphK1−/− saline vs. SphK1−/− LPS treated group (Fig. 5B).

The results of western blot analysis pointed to a significant difference between wild type saline injected and SphK1−/− saline injected animals, thus we tested naïve animals. Supplementary Figure 3 shows significant difference in the expression of GFAP between wild type and SphK1−/− mice (*p<0.0001). Western blot supported immunohistochemical findings. Expression of the astrocyte specific protein GFAP was significantly higher in SphK1−/− naïve animals as compared to wild type (**p = 0.0047) (Fig. S3C).

The combined results of the reactive astrocytes study support our hypothesis that SphK1−/− mice are predisposed to the injury and react even to the injection of the saline by the expression of the glial fibrillary acidic protein. We believe this is a result of SphK1−/− animals being in a state of mild inflammation.

### Loss of Oligodendrocytes Due to LPS Induced Injury

**Figure 6:**
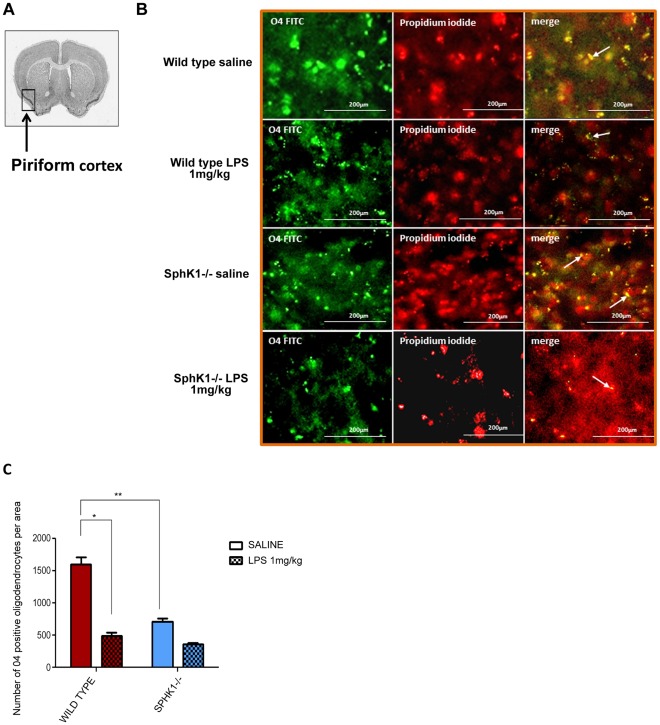
Analysis of cortex area for oligodendrocyte expression. (**A**) Piriform cortex area of the analysis is depicted. Interaural: 4.39; bregma: 0.74. (**B**) Loss of oligodendrocytes (arrows) after intracerebroventricular injection of LPS 1 mg/kg in wild type and SphK1−/− mice. Nuclei were stained with propidium iodide. 40× magnifications are shown. (**C**) Number of oligodendrocytes was calculated per taken area of piriform cortex and analyzed statistically. The significant loss of the oligodendrocytes was determined in wild type group after LPS 1 mg/kg was injected intracerebrally (*p<0.0001) and between wild type saline and SphK1−/saline groups (**p<0.001).

Several studies showed that oligodendrocytes and their progenitors are one of the first types of cells which respond to lipopolysaccharide treatment [7], [46]. Leukoaraiosis is tightly correlated with a loss of oligodendrocytes too [40]. To further characterize the neuroinflammatory effect of LPS in wild type and SphK1−/− mice, we performed immunohistochemical analysis of frozen brain sections using oligodendrocytes progenitors’ specific antibody O4. The principal function of the oligodendrocytes is an insulation of the axons by providing the myelin sheath that is critical for neuronal signal transduction. A single oligodendrocyte cell can expand its processes to fifty axons, thus the integrity of these cells is crucial for a normal function of the brain. Oligodendrocyte progenitor cells are scattered in the brain tissue; however for immunohistochemical analysis the area of the piriform cortex was chosen based on the database of Allen brain atlas of protein expression (Fig. 6A). Loss of oligodendrocytes was noticeable in WT and SphK1−/− animals after LPS treatment (Fig. 6B). Frozen sections of the brains of wild type mice were examined and the number of stained particles was quantified, using Image J software. In the WT group, the number of stained particles declined by 3.2 folds from 1602±113.3 in saline treated animals to 494.3±46.05 in LPS injected mice (Fig. 6C). SphK1−/− mice showed 1.9 folds loss of the myelinating cells from 710±47.83 in saline injected mice to 360±20.98 in LPS injected mice, respectively (Fig. 6B). The significance in loss of oligodendrocytes was reached between wild type saline vs. wild type LPS 1 mg/kg (*p<0.0001) and in wild type saline vs. SphK1−/− saline (**p<0.001) groups. The basal level of quantified oligodendrocytes in mutant controls is 2.3 folds less as compared to the wild type saline treated group (Fig. 6C). These results support our hypothesis that SphK1−/− mice are more susceptible to LPS induced brain injury due to the absence of sphingosine kinase 1, which when present does not allow neuroinflammation to develop to an extreme level.

## Discussion

The primary aim of this study was to determine the pathophysiological changes in the brain as a result of LPS challenge, and to determine the role of sphingosine kinase 1 (SphK1) in these changes. The major finding of the study was that intracerebroventricular injection of LPS (1 mg/kg) induces morphological neuroinflammatory parameters: 1) enlarged lateral ventricles (Fig. 1); 2) distorted brain matter (Fig. 1); 3) increased expression of reactive microglial markers, such as CD68 and ferritin light chain (Fig. 2 and Fig. 3); 4) increases in GFAP expression, the marker of reactive astrogliosis (Fig. 4 and Fig. 5). Also genetic deletion of SphK1 possibly affects neuro and gliogenesis (Figures S2 and S3).

SphK1−/− mice expressed increased inflammatory damage to the brain induced by LPS as compared to wild type. The increased neuroinflammation induced by LPS injection suggests that absence of SphK1 gene and the consequent alteration of the sphingolipid signaling pathway are responsible for the increased inflammatory damage observed in the brains of SphK1−/− mice. This corresponds with previous studies that showed that SphK1 triggers both the inflammatory response as well as LPS-activated apoptosis [23], [29], [31], [47]. The current study shows that the presence of SphK1 is essential for maintaining neuroinflammatory process under certain control.

Studies have shown a close correlation of S1P levels with the expression of SphK1 and SphK2. Treatment with a SphK1 siRNA down-regulated serum levels of S1P in collagen-induced arthritis mouse model [48]. Lower S1P levels were reported in mice that had homozygous deletions of either SphK1 or SphK2 [49].

A variety of markers of brain injury show increased neuroinflammatory damage in SphK1−/− mice. White matter rarefactions are increased in SphK1−/− mice intracerebrally injected with LPS (Fig. 1). Development of rarefactions results from the loss of white matter [7], [50] and is associated with neuroinflammation [39], oligodendrocyte loss and demyelination [40]. In the current study, greater neuroinflammatory damage to white matter in SphK1−/− mice injected with LPS was likely the result of increased rarefactions and loss of O4+ oligodendrocyte progenitor cells (Fig. 1 and Fig. 6). During neuroinflammation, TNFα can induce oligodendrocytes loss [51]; the reduction of TNFα levels in SphK1−/− animals may underlie why fewer oligodendrocytes are lost following LPS injection [46], [51]. In addition to activating caspases, TNFα activates the transcription factor NFκB, which is a potent survival signal. SphK may directly change TNFα signaling toward the activation of NFκB through direct binding of SphK1 with receptor associated factor (TRAF2) [52].

Edema leading to the enlargement of the lateral ventricles of the brain is another hallmark of neuroinflammation. LPS loosens tight junctions, and as a result cerebral ventricles enlarge [36], [37]. Interestingly, the ventricular enlargement is also seen in control saline intracerebrally injected SphK1−/− mice and in naïve SphK1−/− animals (Fig. 1 and Fig. S2). This suggests that SphK1 regulates the blood brain barrier (BBB) in the absence of neuroinflammation. The role of S1P is well established in the functions of vascular endothelium [53], [54]. Lowered S1P levels are hypothesized to derange cellular metabolism that leads to impaired endothelial function and ventricular edema. In particular, earlier studies by Peng et al., (2004) and McVerry et al., (2004) have shown that infusion of S1P significantly decreases pulmonary/renal vascular leakage and inflammation in a murine model of LPS-mediated acute lung injury, which may represent a novel therapeutic strategy for vascular barrier dysfunction. Furthermore, exogenous S1P added to cultured neonatal rat ventricular myocytes was protective against hypoxia-induced cell death. Similarly, in an *ex vivo* mouse model, S1P administration via aortic cannula before ischemia/reperfusion injury lead to improved hemodynamics, reduced creatine kinase release, and diminished infarct size. These results clearly suggest the mechanism, which is common in preventing vascular leak and suggesting the role for sphingosine kinases. However, if this is true or not in brain tissue is not clear, and the effects of LPS induced brain injury is rarely studied. If synthesis of S1P is highly dependent on its phosphorylating enzyme SphK1, the functions of the end product highly depend on the functions of the kinase. S1P stimulation induces endothelial cell proliferation, migration and survival [55], thus it is not a surprise that in our study we show a protection provided by SphK1 on the extent of neuroinflammation, which develops as a result of LPS injection into the ventricles of the brain. In addition, an interesting study where bone marrow derived progenitor cells (BMPC) were used to improve LPS induced pulmonary edema revealed that BMPC from SphK1−/− mice did not provide similar protection [56].

Usually, neuroinflammation is divided into acute and chronic. Acute is considered to be somewhat beneficial, because it tends to minimize further injury and contribute to the repair of damaged tissue. It includes activation of the resident immune cells (microglia) resulting in a phagocytic phenotype and the release of inflammatory mediators such as cytokines and chemokines. In contrast, chronic inflammation persists long after an initial injury or insult. Microglia are activated for much longer period of times, thus release of cytokines is longstanding. In addition, chronic inflammation results in increased oxidative, nitrosative stress, development of the leukoaraiosis, a loss of brain matter that usually occurs as a result of ischemic injury [40], [57] and is connected to inflammation [39]. All these factors continue the inflammatory cycle, activate additional microglia, promoting their proliferation and result in further release of inflammatory factors.

Analysis of microglial activation provides additional evidence that SphK1 negatively regulates neuroinflammation. LPS activates microglia by binding to its receptor TLR-4 that is abundantly present in the microglial membrane [58], [59]. Microglia in the brain are potently activated by LPS injected either intraperitoneally or intracerebrally, as seen using two markers of microglial activation: CD68 and ferritin light chain (Fig. 2 and Fig. 3). Since the BBB is likely compromised by LPS, it is not clear whether the activated microglias are resident to the brain, or are from the plasma [60]. Regardless of their origin, microglial activation is greatly increased in SphK1−/− mice. Activated microglia can kill oligodendrocytes. Therefore, increased microglial activation in SphK1−/− mice may underlie the reduction in oligodendrocytes and the development of white matter rarefactions.

Further evidence that SphK1 is a negative regulator of inflammation comes from the analysis of glial activation and cytokine expression [61]. Astrocytes of SphK1−/− mice express enhanced levels of glial fibrillary acidic protein (GFAP) (Fig. 4 and Fig. 5). Increased astroglial expression of GFAP is frequently associated with activated microglia and elevated expression of proinflammatory cytokines such as TNFα and IL-6 [34], [44], [51]. LPS treatment induces microglia and astrocytes to produce large amounts of proinflammatory cytokines and chemokines [46], [62]. Proinflammatory cytokines and chemokines trigger the sphingolipid signaling pathway leading to the production of pro-survival S1P [20], [63]. In astrocytes, S1P receptor activation leads to the activation of extracellular signal-regulated kinase 1/2, which results in cellular proliferation and neurotrophic factor production as well as platelet derived growth factor (PDGF) production [64]. It is likely that there is increased production of proinflammatory cytokines and chemokines in SphK1−/− mice which leads to increased astrocyte activation.

Miron’s study also has found that the synthetic analog of sphingosine (Fingolimod) FTY720, which was approved as a sphingosine-1-phosphate receptor modulator in multiple sclerosis, induced remyelination and astrogliosis and that the action was mediated via the S1P3 and S1P5 receptors, particularly through the S1P5 which is highly expressed on mature oligodendrocytes. SphK1−/− mice have fewer oligodendrocyte precursors than wild-type mice (Fig. 6). SphK1 is essential for brain development [49]. In our study, the absence of SphK1 may lead to altered oligodendrocyte development. Alternatively, the finding of fewer oligodendrocyte precursors in SphK1−/− mice may result from an increased rate of apoptosis in these cells resulting in a higher susceptibility of the CNS to LPS induced injury. It was shown earlier that during neuroinflammation there is a certain loss of oligodendrocytes [46]. Another study showed that TNFα mediates loss of oligodendrocytes [51]. There are reports of a direct interaction of SphK with TNF receptor associated factor (TRAF2), suggesting the role of SphK in NFκB activation [52]. It is proposed that TNFα-induced activation of NF_K_B and JNK most likely bifurcates at the site of TRAF2 binding. While SphK-mediated TRAF2 promotes NFκB activation, JNK activation is SphK-independent. Furthermore, we identified an inducible interaction between Lyn kinase and SphK1 localized to lipid rafts (data not shown). We demonstrated that during TNFR receptor signaling SphK1 association with TNFR complex and its translocation to the lipid rafts plays an important role in SphK activation and S1P synthesis. SphK1 is potently anti-apoptotic [65] suggesting that SphK1 prevents apoptosis of oligodendrocytes in the absence of LPS.

In the last few years, studies of sphingosine kinases have generated a lot of controversy. For instance, a study published in 2006 showed that SphK1 is not required for inflammatory cell recruitment during thioglycollate-induced peritonitis [66]. On the other hand, numerous *in vivo* and *in vitro* studies where sphingosine kinases were inhibited pharmacologically have demonstrated a role of SphKs in triggering the inflammatory response, and that they are responsible for LPS activated apoptosis [23], [29], [31], [47]. We believe that sphingosine kinase 1, a so-called pro-survival enzyme, [67], [68] can function as a modulator of inflammatory processes.

The presented data in this manuscript provides strong evidence that SphK1 regulates neuroinflammation produced by a strong inducer of inflammation, such as LPS. [67], [68]. SphK1 also regulated a neuroinflammatory like process in the absence of LPS. SphK1 action is widespread in the brain with effects on the vascular epithelium, astrocytes and microglia, and SphK1 is essential for controlling the neuroinflammatory process.

This data along with earlier studies will be taken into consideration for a further investigation of the possible mechanisms behind the development of the LPS-induced neuroinflammation and observed parameters such as leukoaraiosis, gliosis, and loss of oligodendrocytes.

## Supporting Information

Figure S1 **Construction of the imaginary triangle inside the lateral ventricle for the size quantification.** Sizes of both lateral ventricles of the brain were measured as areas of a triangle (A = ½ of the base × height). Total area of the brain section was taken as a 100 percent and percent taken by lateral ventricles was calculated.
(TIF)

Figure S2 **Hematoxylin and eosin staining of naïve wild type and SphK1−/− animal’s brain slices.** **(A)** Enlarged ventricles (stars) are shown in SphK1−/− animals. Magnifications of 4× and 40× are shown. **(B)** Table represents mean ± SEM (n = 10) of the areas of lateral ventricles of the brains of wild type and SphK1−/− mice. **(C)** Significant difference was found between wild type and SphK1−/− mice, *p = 0.0008.
(TIF)

Figure S3 **(A)** **Immunohistochemical analysis of astrocyte specific protein GFAP in wild type and SphK1−/− naïve mice.** **(B)** Statistical analysis of the expression of GFAP in a series of images (n = 6); *p<0.0001. **(C)** Western Blot analysis of the GFAP protein, expressed by astrocytes. **(D)** Significant difference was found in comparison of wild type vs. SphK1−/− (n = 4); *p = 0.0047.
(JPEG)
