# Supplementary figures and images for: Sphingosine Kinase 1 Deficiency Exacerbates LPS-Induced Neuroinflammation
Source: PLoS One. 2012 May 17;7(5):e36475. doi: 10.1371/journal.pone.0036475 (PMC3355156; doi:10.1371/journal.pone.0036475)

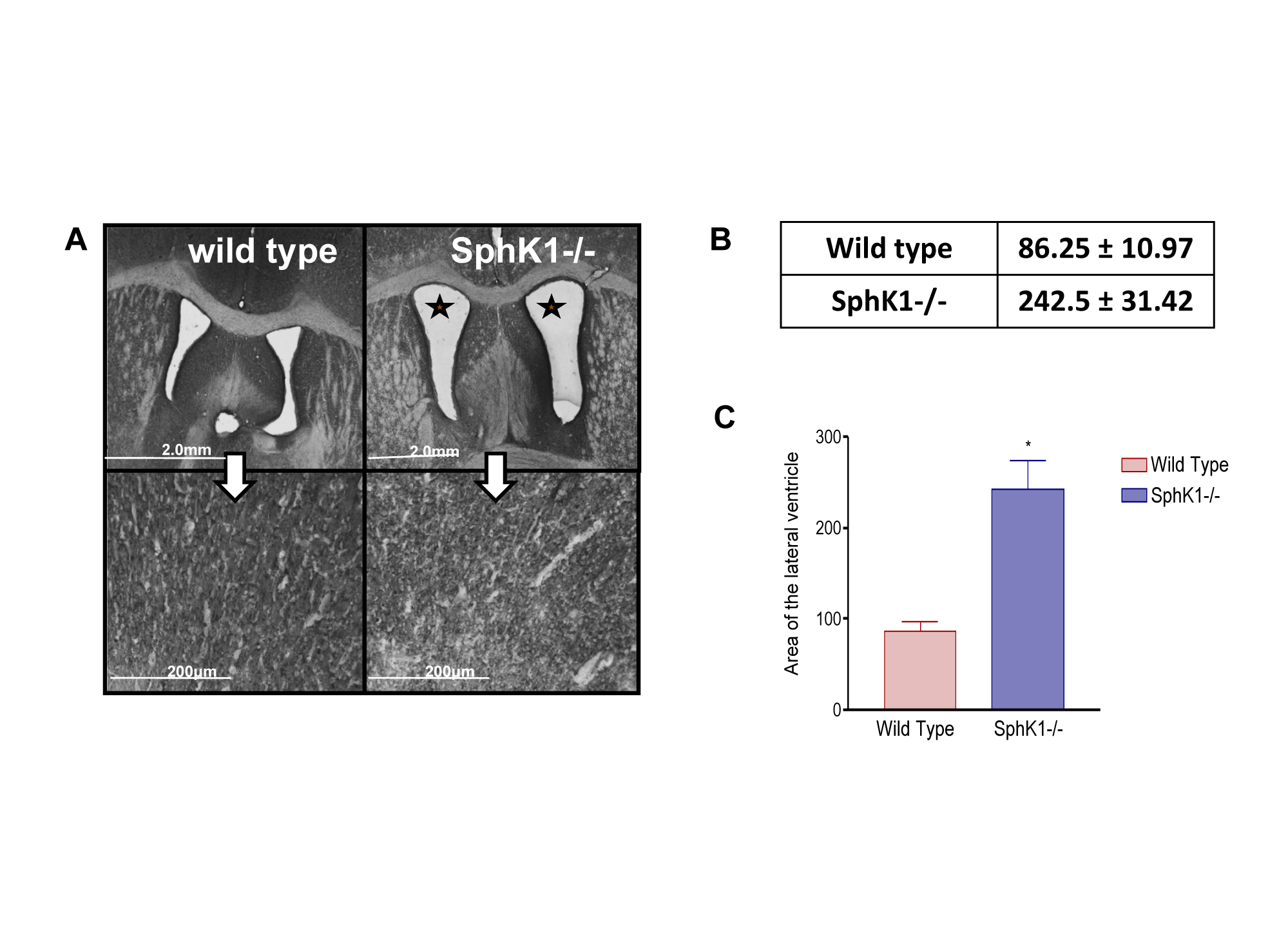

Supplement: Figure S1 — Construction of the imaginary triangle inside the lateral ventricle for the size quantification. Sizes of both lateral ventricles of the brain were measured as areas of a triangle (A = ½ of the base × height). Total area of the brain section was taken as a 100 percent and percent taken by lateral ventricles was calculated. (TIF) [file pone.0036475.s001.tif]

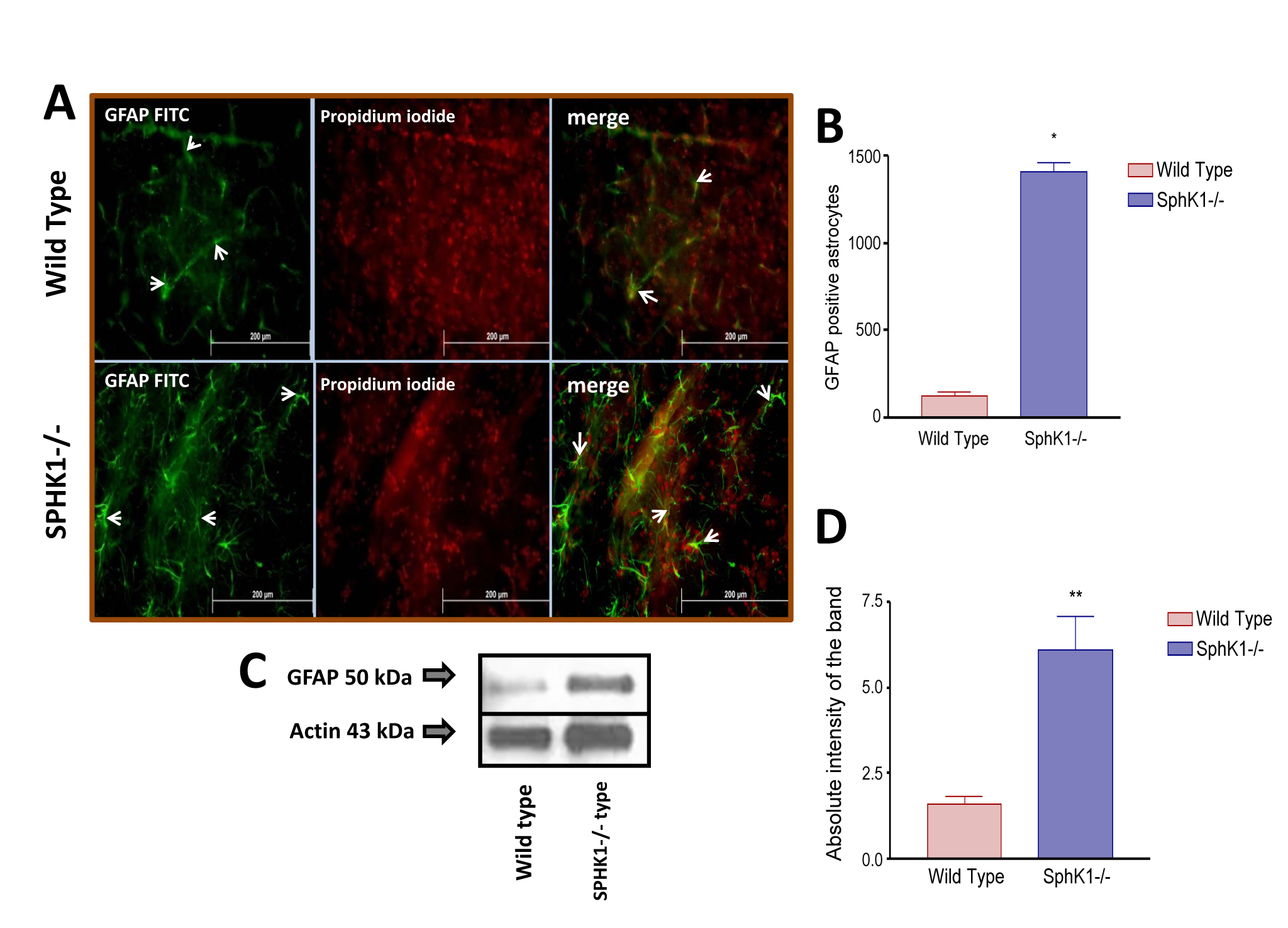

Supplement: Figure S2 — Hematoxylin and eosin staining of naïve wild type and SphK1−/− animal’s brain slices. (A) Enlarged ventricles (stars) are shown in SphK1−/− animals. Magnifications of 4× and 40× are shown. (B) Table represents mean ± SEM (n = 10) of the areas of lateral ventricles of the brains of wild type and SphK1−/− mice. (C) Significant difference was found between wild type and SphK1−/− mice, *p = 0.0008. (TIF) [file pone.0036475.s002.tif]

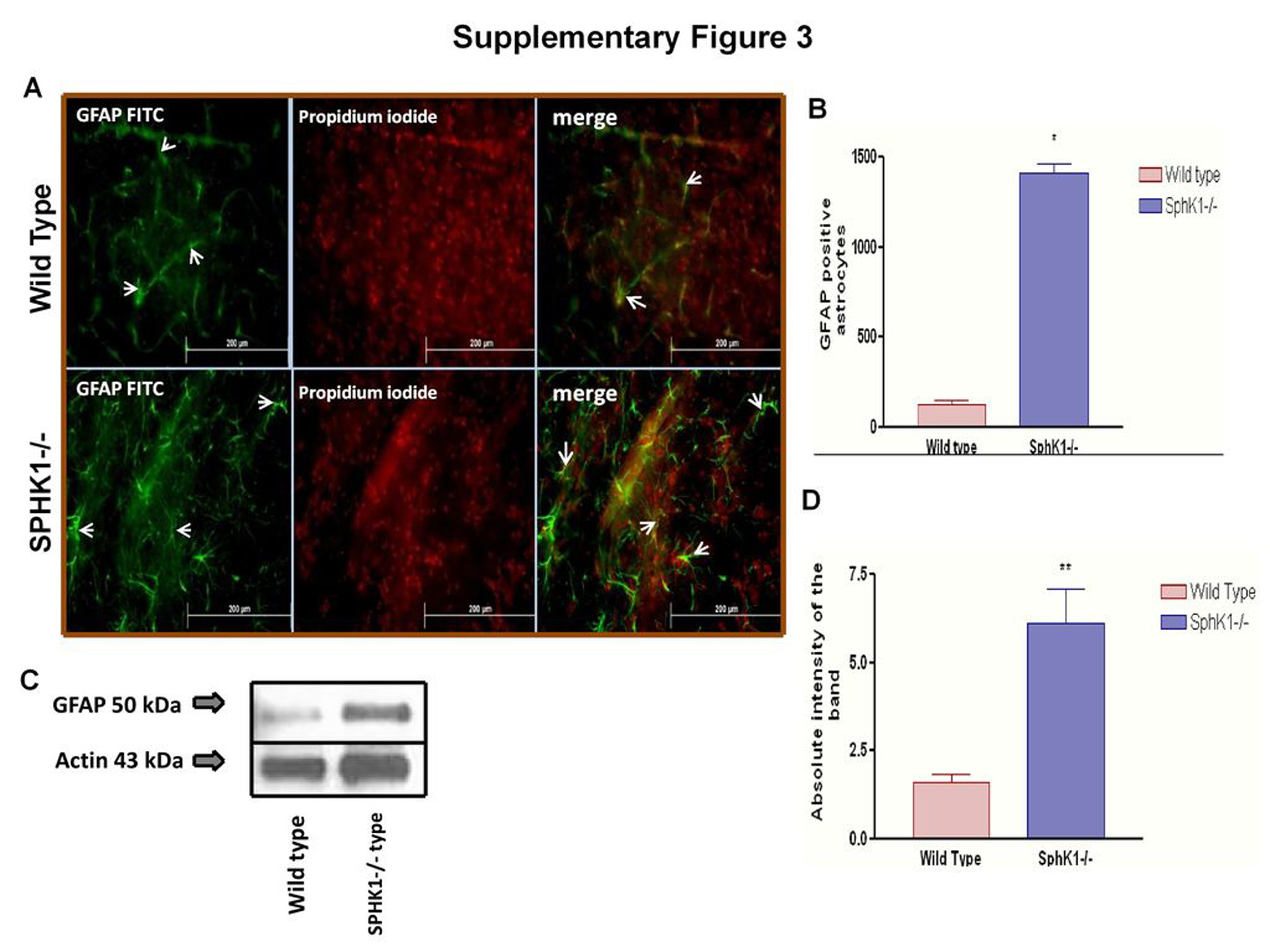

Supplement: Figure S3 — (A) Immunohistochemical analysis of astrocyte specific protein GFAP in wild type and SphK1−/− naïve mice. (B) Statistical analysis of the expression of GFAP in a series of images (n = 6); *p<0.0001. (C) Western Blot analysis of the GFAP protein, expressed by astrocytes. (D) Significant difference was found in comparison of wild type vs. SphK1−/− (n = 4); *p = 0.0047. (JPEG) [file pone.0036475.s003.jpeg]
